# Resveratrol promotes osteogenesis and alleviates osteoporosis by inhibiting p53

**DOI:** 10.18632/aging.103262

**Published:** 2020-05-27

**Authors:** Tao Yu, Zaiyan Wang, Xiaomeng You, Haichao Zhou, Wenbao He, Bing Li, Jiang Xia, Hui Zhu, Youguang Zhao, Guangrong Yu, Yuan Xiong, Yunfeng Yang

**Affiliations:** 1 Department of Orthopedic Surgery, Tongji Hospital, Tongji University School of Medicine, Shanghai 200065, China; 2 Department of Respiratory Medicine, Shanghai University of Medicine and Health Sciences Affiliated Zhoupu Hospital, Shanghai 201318, China; 3 Department of Orthopedic Surgery, Brigham and Women’s Hospital, Harvard Medical School, Boston, MA 02115, USA; 4 Department of Orthopedics, Union Hospital, Tongji Medical College, Huazhong University of Science and Technology, Wuhan 430022, China

**Keywords:** osteoporosis, resveratrol, KEGG pathways, bone mass density, bioinformatics analysis

## Abstract

Although osteoporosis is one of the most common chronic age-related diseases, there is currently no gold standard for treatment. Evidence suggests resveratrol, a natural polyphenolic compound, may be helpful in the treatment of osteoporosis and other diseases. However, the molecular mechanisms underlying the anti-osteoporotic effects of resveratrol remain largely unknown. In the present study, KEGG pathway enrichment analysis of resveratrol-targeted genes identified 33 associated pathways, 12 of which were also involved in osteoporosis. In particular, the MDM2/p53 signaling pathway was identified as a potential key pathway among the shared pathways. *In vitro* experiments indicated that MDM2-mediated p53 degradation induced osteoblast differentiation, and resveratrol could partially reverse p53-dependent inhibition of osteogenic differentiation. These findings suggest resveratrol may alleviate osteoporosis at least in part by modulating the MDM2/p53 signaling pathway.

## INTRODUCTION

Osteoporosis (OP) is a common metabolic bone disease in postmenopausal women and the elderly [1]. The pathological characteristics of osteoporosis include abnormal bone microarchitecture, decreased bone density, and increased bone fragility, which result in an increased risk of fractures [2]. An estimated 200 million people currently suffer from osteoporosis worldwide, and about 34 million patients in the USA have been diagnosed with osteoporosis or low bone mass [3]. Osteoporosis can cause pain, spinal deformities, and fragile fractures. Fragile fractures are usually caused by low-energy impacts, such as a fall from standing height, a slight collision, or other routine minor injuries. In a 50-year-old woman, the risk of developing osteoporotic fractures may be has high as 50% [4]. Vertebral and hip fractures are two common types of osteoporotic fractures. Vertebral fractures can cause long-term pain and severely affect the quality of life, while hip fractures can prevent patients from standing or walking and can even increase the risk of death [4]. Osteoporosis has thus become a major public health problem worldwide, placing heavy economic burdens on both patients and healthcare systems [3]. There is currently no gold standard treatment for osteoporosis.

Resveratrol (3,5,4′-trihydroxy-trans-stilbene) is a natural polyphenolic compound found in several plants, including grapes, berries, and peanuts [5, 6]. Growing evidence shows that resveratrol has various biological effects [7–9], including protective effects in osteoporosis. For example, bone mass density (BMD) reduction and microarchitectural deterioration were reversed by resveratrol in a rodent model [8]. Resveratrol may impact bone homeostasis by acting as both an anabolic and antiresorptive agent [10]. However, the detailed mechanisms underlying the effects of resveratrol on bone health remain unclear.

Bioinformatics, which uses computational techniques to organize and integrate experimental data, has been used to great effect in many studies [11, 12]. In this study, Kyoto Encyclopedia of Genes and Genomes (KEGG) pathways associated with both resveratrol-targeted genes and osteoporosis progression were analyzed using bioinformatic tools. The p53 signaling pathway was identified as a key KEGG pathway involved in the protective effects of resveratrol on osteoporosis. We therefore performed an *in vitro* study to further examine whether resveratrol could counteract the negative effects of p53 on MDM2-mediated osteogenic differentiation.

## RESULTS

### Resveratrol-targeted genes and the interaction network

**Figure 1:**
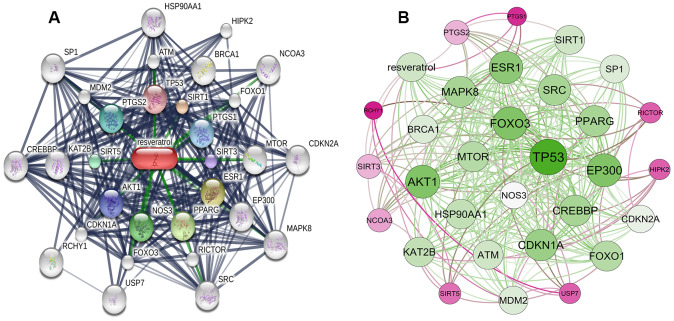
**Construction of resveratrol-targeted genes interaction network.** (**A**) Interaction network constructed using STITCH. First shell (chemical-protein): TP53, SIRT1, PTGS1, SIRT3, ESR1, PPARG, NOS3, AKT1, SIRT5, PTGS2. Second shell (protein-protein): ATM, BRCA1, FOXO1, MTOR, EP300, RICTOR, FOXO3, CDKN1A, KAT2B, MDM2. Third shell (protein-protein): HSP90AA1, HIPK2, NCOA3, CDKN2A, MAPK8, SRC, USP7, RCHY1, CREBBP, SP1. (**B**) Weighted interaction network indicating that TP53 had the highest weight.

In total, 30 resveratrol-targeted genes were identified in Search Tool for Interacting Chemicals (STITCH) using a three shell limit. Interaction networks among resveratrol-targeted genes were then constructed in STITCH (Figure 1A). TP53, SIRT1, PTGS1, SIRT3, ESR1, PPARG, NOS3, AKT1, SIRT5, and PTGS2 were identified as members of the first shell, indicating that resveratrol might directly affect these genes. The second shell consisted of ATM, BRCA1, FOXO1, MTOR, EP300, RICTOR, FOXO3, CDKN1A, KAT2B, and MDM2, and the third shell included HSP90AA1, HIPK2, NCOA3, CDKN2A, MAPK8, SRC, USP7, RCHY1, CREBBP, and SP1, indicating that resveratrol might have secondary effects on these genes. A network visualization constructed based on interaction weights indicated that TP53 had the highest weight of all of these genes (Figure 1B).

### Identification of KEGG pathways associated with osteoporosis and resveratrol-targeted genes

**Figure 2:**
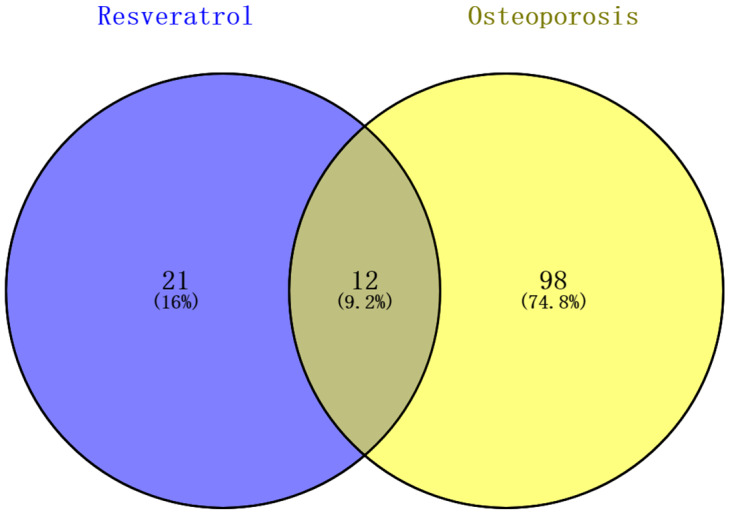
**Identification KEGG pathways associated with both resveratrol-target genes and osteoporosis.** 33 KEGG pathways associated with resveratrol-target genes and 110 associated with osteoporosis were identified; 12 (9.2%) KEGG pathways associated with both are shown in the Venn diagram.

Enrichment analysis of the resveratrol-targeted genes using Database for Annotation, Visualization, and Integrated Discovery (DAVID) identified 33 KEGG pathways with p <0.05. 110 KEGG pathways involved in human osteoporosis were retrieved using miRWalk2.0. Twelve KEGG pathways associated with both osteoporosis and resveratrol-targeted genes were identified and visualized using a Venn Diagram online tool (Figure 2). Among them, the five KEGG pathways with smallest p values were prostate cancer pathway, pathway in cancer, glioma pathway, p53 signaling pathway, and cell cycle signaling pathway (Table 1).

**Table 1:** Top five KEGG pathways and associated genes.

**Term**	**KEGG pathway**	**Icariin-target genes**	***P*-value**
hsa05215	Prostate cancer pathway	AKT1, CDKN1A, HSP90AA1, EP300, CREBBP, TP53, FOXO1, MDM2, MTOR	9.5E-10
hsa05200	Pathways in cancer	HSP90AA1, PTGS2, PPARG, CREBBP, TP53, FOXO1, AKT1, CDKN1A, EP300, CDKN2A, MDM2, MAPK8, MTOR	8.1E-9
hsa05214	Glioma pathway	AKT1, CDKN1A, CDKN2A, TP53, MDM2, MTOR	4.4E-6
hsa04115	p53 signaling pathway	CDKN1A, CDKN2A, TP53, MDM2, RCHY1, ATM	5.2E-6
hsa04110	Cell cycle signaling pathway	CDKN1A, CDKN2A, EP300, CREBBP, TP53, MDM2, ATM	6.6E-6

### Identification of hub genes

**Figure 3:**
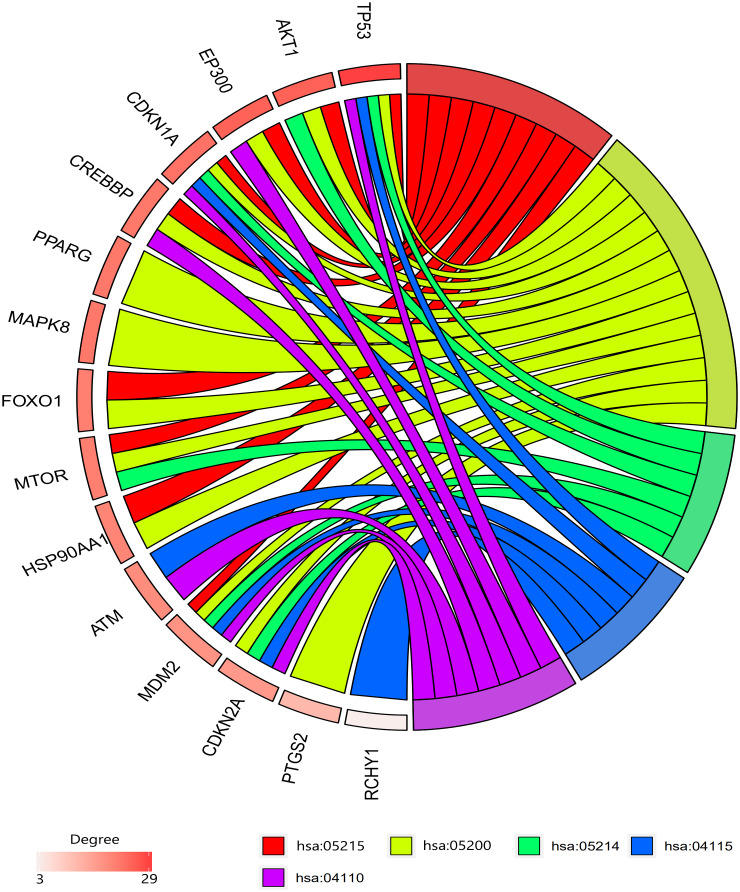
**Gene enrichment analysis results. TP53, CDKN1A, and MDM2 were involved in all top five pathways.** TP53, AKT1, and EP300 had the highest degrees. Hsa05215: prostate cancer pathway, hsa05200: pathway in cancer, hsa05214: glioma pathway, hsa04115; p53 signaling pathway, hsa04110: cell cycle signaling pathway.

**Figure 4:**
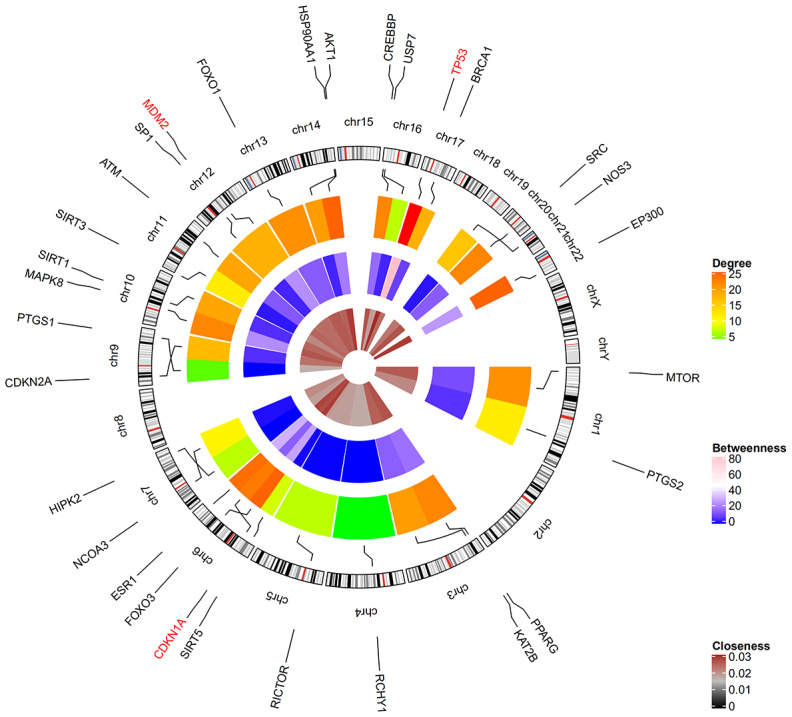
**Circular visualization of chromosomal positions and connectivity of resveratrol-target genes.** Gene names are shown in the outer circle. Different colors represent different degree, betweenness, and closeness values. The outer circle represents chromosomes; lines connect each gene to its chromosomal location. The three hub genes TP53, CDKN1A, and MDM2 highlighted in red are located in chr17, chr6, and chr12, respectively.

Among the 30 resveratrol-targeted genes, TP53, AKT1, EP300, CDKN1A, CREBBP, PPARG, MAPK8, FOXO1, MTOR, HSP90AA1, ATM, MDM2, CDKN2A, PTGS2, and RCHY1 were involved in the top five shared KEGG pathways (Figure 3). TP53, CDKN1A, and MDM2, which were involved in all top five KEGG pathways, were identified as hub genes. A circular visualization of chromosomal positions and connectivity of resveratrol-target genes is shown in Figure 4. Degree, betweenness, and closeness were highest for TP53.

### Retrieval of KEGG pathways related to resveratrol-targeted genes

**Figure 5:**
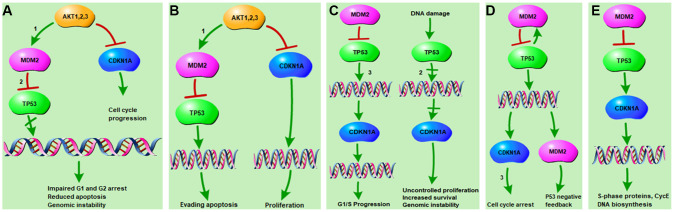
**KEGG pathways related to resveratrol-targeted genes.** (**A**) Resveratrol-targeted genes related to the Prostate cancer pathway. (1) The PI3K-Akt signaling pathway and (2) p53 signaling pathways are associated with impaired G1/G2 arrest, reduced apoptosis, and genomic instability. (**B**) Resveratrol-targeted genes related to pathways in cancer. (1) The PI3K-Akt signaling pathway is associated with apoptosis evasion and proliferation. (**C**) Resveratrol-targeted genes related to the glioma pathway. (2) The p53 signaling pathway and (3) cell cycle pathway are associated with G1/S progression, uncontrolled proliferation, increased survival, and genomic instability. (**D**) Resveratrol-targeted genes related to the p53 signaling pathway. (3) The cell cycle pathway is associated with cell cycle arrest and p53 negative feedback. (**E**) Resveratrol-targeted genes related to the cell cycle signaling pathway, which is associated with biosynthesis of S-phase proteins and CycE DNA.

The downstream cellular responses of the top five KEGG pathways associated with both osteoporosis and resveratrol-targeted genes are shown in Figure 5. They include G1/G2 arrest, cellular apoptosis, genomic instability, proliferation, G1/S progression, survival, and cell cycle arrest. These results indicate that resveratrol impacts a variety of cellular activities through the PI3K, p53, and cell cycle signaling pathways.

### MDM2-mediated p53 degradation induces osteoblast differentiation *in vitro*

**Figure 6:**
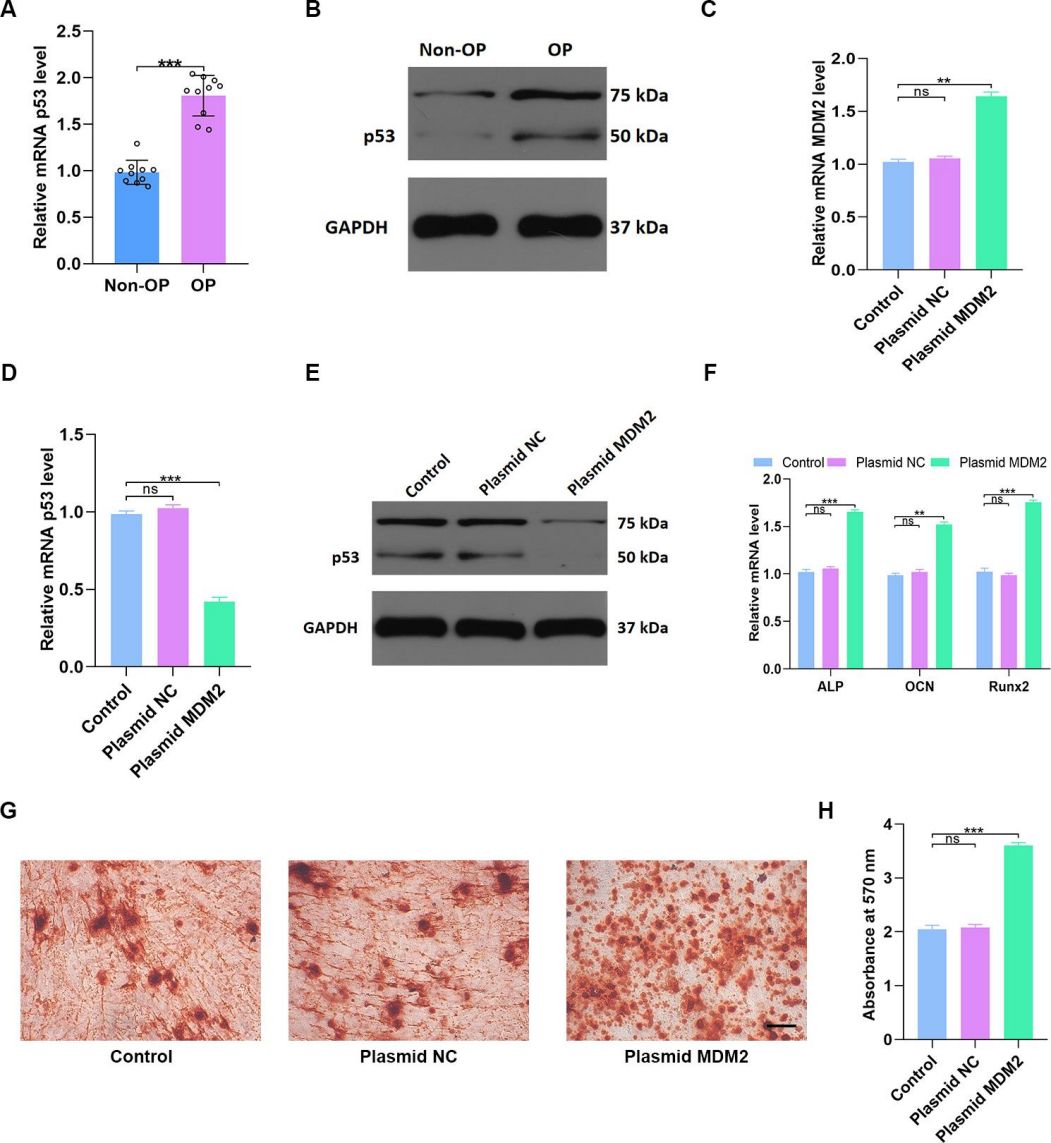
**MDM2-mediated p53 degradation induces osteoblast differentiation *in vitro*.** (**A**, **B**) p53 levels in non-OP patients and OP patients were measured by qRT-PCR and western blot; n=10 per group. (**C**) MDM2 expression in hMSCs was assessed by qRT-PCR analysis after different treatments. (**D**–**E**) p53 levels were measured by qRT-PCR and western blot in the three groups. (**F**) Osteogenic gene levels were measured by qRT-PCR. (**G**–**H**) Alizarin red-mediated calcium staining in hMSCs 21 days after transfection with different constructs. Scale bar = 10mm. Data are means ± SD. *p < 0.05, **p < 0.01, ***p < 0.001.

p53 gene and protein expression were measured in non-OP (n=10) and OP patients (n=10) using qRT-PCR and western blotting, respectively. p53 gene and protein expression were significantly higher in OP patients (p < 0.001, Figure 6A, 6B) than in non-OP patients. Transfection of human mesenchymal stem cells (hMSCs) with an MDM2 overexpression plasmid successfully increased MDM2 gene expression compared to untreated cells (Control) and empty vector transfection (Plasmid NC) (Figure 6C). As expected, p53 gene and protein expression decreased significantly in hMSCs transfected with MDM2 plasmid (p < 0.001 Figure 6D, 6E). In addition, expression of the osteogenic genes ALP OCN and Runx2 also increased significantly in MDM2 plasmid-treated hMSCs compared to untreated control and empty vector-treated cells (p < 0.001 Figure 6F). Alizarin red-mediated calcium staining also showed enhanced mineral deposition in the MDM2 plasmid group (p < 0.001 Figure 6G, 6H). These results indicated that MDM2 induced p53 degradation and promoted osteogenesis in hMSCs.

### Resveratrol partially reverses p53-induced inhibition of osteogenic differentiation

**Figure 7:**
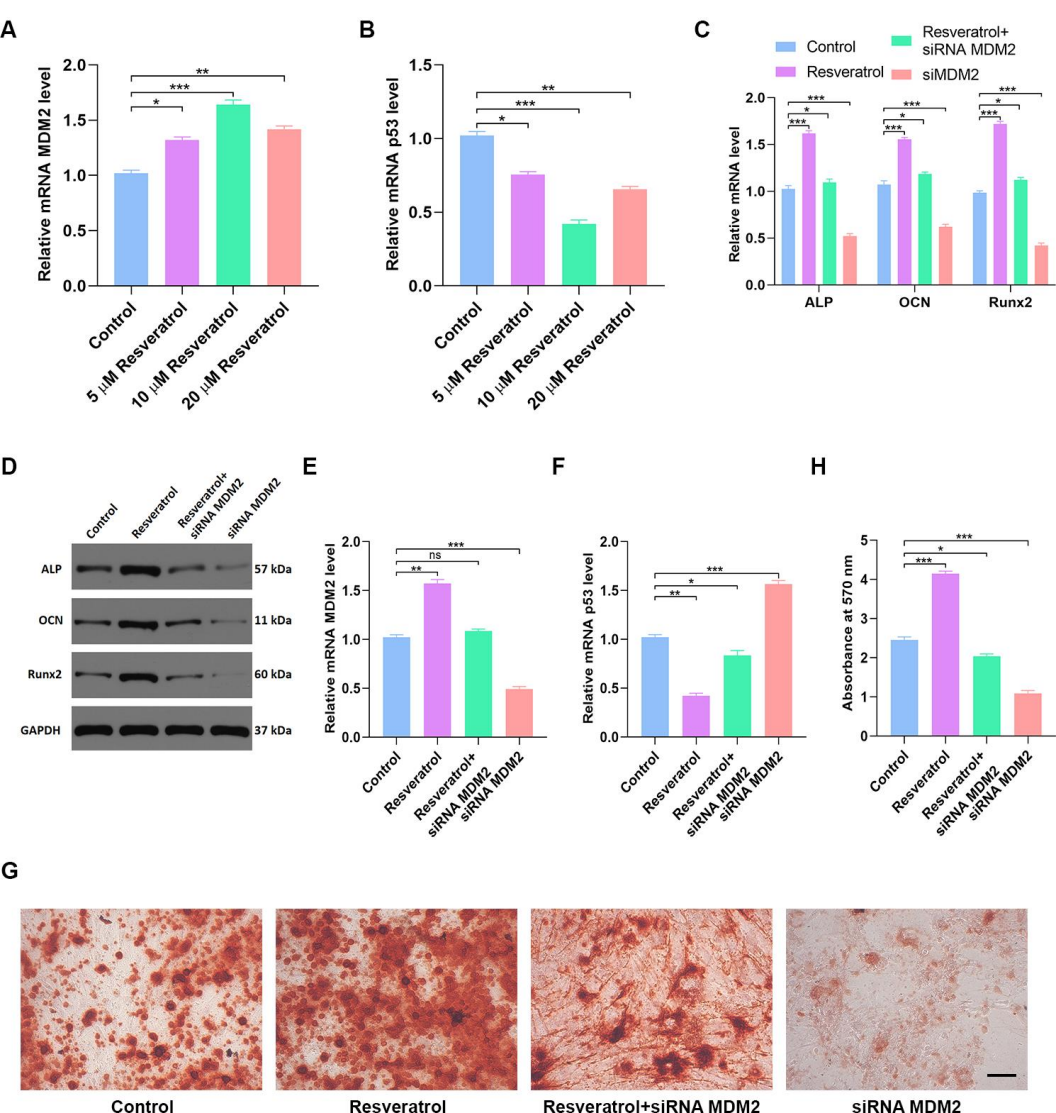
**Resveratrol partially reverses p53-induced inhibition of osteogenic differentiation.** (**A**) Effects of different concentrations of resveratrol on MDM2 expression were measured by qRT-PCR. (**B**) p53 expression in different groups was assessed by qRT-PCR. (**C**, **D**) Osteogenic genes expression in different groups was measured by qRT-PCR and western blot. (**E**) MDM2 expression in hMSCs after different treatments was assessed by qRT-PCR. (**F**) p53 expression in different groups was assessed by qRT-PCR. (**G**–**H**) Alizarin red-mediated calcium staining in hMSCs 21 days after transfection with different constructs. Scale bar = 10mm. Data are means ± SD. *p < 0.05, **p < 0.01, ***p < 0.001.

The effects of different concentrations of resveratrol on MDM2 expression were measured by qRT-PCR. 10 μM resveratrol induced both the largest increase in MDM2 gene expression (Figure 7A) and the largest decrease in p53 expression (Figure 7B). As expected, ALP, OCN, and Runx2 expression were significantly increased by resveratrol treatment (Figure 7C–7D). Moreover, MDM2 gene expression was significantly higher (Figure 7E), while p53 expression was significantly lower (Figure 7F), in hMSCs treated with resveratrol than in the other groups. Furthermore, alizarin red-mediated calcium staining showed enhanced mineral deposition in the resveratrol group (p < 0.05 Figure 7G–7H), indicating that resveratrol might increase mineral bone mass. In summary, these results suggest that resveratrol promotes osteogenesis by inhibiting the p53 signaling pathway.

## DISCUSSION

Although osteoporosis is a widespread disease [13] that has become a major challenge for public health systems worldwide [3], there is currently no gold standard treatment. While resveratrol can reverse both osteoporosis-associated BMD reduction and microarchitectural deterioration [8], the mechanisms responsible for these effects remain unknown. In this study, bioinformatics analyses and *in vitro* studies were performed to investigate the molecular mechanisms of resveratrol’s effects on osteoporosis pathologies.

In this study, 12 KEGG pathways associated with both osteoporosis and resveratrol-targeted genes were identified using bioinformatics tools. Among these, the top five KEGG pathways with the smallest p values were prostate cancer pathway, pathway in cancer, glioma pathway, p53 pathway, and cell cycle pathway. Resveratrol-targeted genes were associated with G1/G2 arrest, apoptosis, and genomic instability in the prostate cancer pathway (Figure 5A), apoptosis and proliferation in pathway in cancer (Figure 5B), G1/S progression, proliferation, survival and genomic instability in the glioma pathway (Figure 5C), cell cycle arrest and p53 feedback in the p53 pathway (Figure 5D), and S-phase proteins and CycE DNA biosynthesis in the cell cycle pathway (Figure 5E). Thus, resveratrol-targeted genes exerted biological effects primarily through the p53 signaling pathway. p53 inhibits cancer development and progression via several mechanisms, including apoptosis, regulation of DNA replication, cell division, and inhibition of angiogenesis [14, 15]. The p53 protein is encoded by the TP53 gene, which was identified in this study as the hub gene with the highest degree of interaction in the network. TP53, CTNNB1, and SP1 modulate the expression of most of the differentially expressed genes that are upregulated and play important roles in primary osteoporosis [16]. Fu Jia et. al. demonstrated that pri-miR-34b/c rs4938723 and TP53 Arg72Pro polymorphisms may contribute to the risk of osteoporosis [17]. In this study, both qRT-PCR and western blots indicated that p53 was enriched in osteoporosis (Figure 6A, 6B). In addition, qRT-PCR and Alizarin-red staining showed that MDM2-mediated inhibition of p53 induced osteoblast differentiation *in vitro* (Figure 6C–6G), indicating that p53 promoted the pathological progression of osteoporosis.

Resveratrol (3,5,4′-trihydroxy-trans-stilbene) is a natural polyphenolic compound found in several plants [5, 6]. Accumulating evidence shows that resveratrol has anti-inflammatory, antioxidant, and other protective effects in osteoporosis and in aging-induced cognitive impairment [7–9]. Ali Mobasheri and Mehdi Shakibaei reported that resveratrol can modulate bone cell metabolism and bone turnover due to its osteogenic and osteoinductive properties [18, 19]. In this study, resveratrol partially reversed p53-induced inhibition of osteogenic differentiation in *in vitro* experiments (Figure 7). These results indicate that resveratrol may protect against osteoporosis by inhibiting the p53 signaling pathway.

Some limitations in this study should be considered when interpreting the results. Firstly, the effects of different durations of resveratrol treatment were not investigated. Furthermore, potential differences in p53 enrichment in different osteoporosis subtypes were not examined. Finally, additional studies are needed to compare the effects of resveratrol on osteoporosis development and progression with those of other drugs.

In conclusion, bioinformatics analysis revealed that the protective effects of resveratrol against osteoporosis were associated with its interaction with the prostate cancer pathway, pathway in cancer, glioma pathway, p53 signaling pathway, and cell cycle signaling pathway. Our *in vitro* experiment further indicated that resveratrol exerts anti-osteoporosis effects by inhibiting the p53 signaling pathway, and may thus serve as a promising osteoporosis treatment.

## MATERIALS AND METHODS

### Construction of the resveratrol-targeted genes interaction network

The STITCH database integrates data on the effects of over 430000 chemicals on gene expression [20]. Resveratrol-targeted genes were identified using the default settings in STITCH, and the STITCH online tool was used to construct an interaction network for resveratrol and its targeted genes. Degree, betweenness, and closeness were analyzed for each gene in the network and visualized using Cytoscape 3.7.2. Degree represents the extent to which one node is associated with all the other nodes in the network, closeness represents how far one node is from other nodes in the network, and betweenness is the number of times a node acts as the shortest bridge between two other nodes. These measures for all resveratrol-targeted genes were then imported into Gephi software and a weighted interaction network was constructed.

### Shared KEGG pathways involved both in osteoporosis and resveratrol-targeted genes

miRWalk2.0, a comprehensive archive containing the largest existing collection of predicted and experimentally verified miRNA-target gene interactions [21], was used to identify human osteoporosis-associated KEGG pathways. DAVID, an integrated biological knowledgebase with analytic tools for systematically extracting biological meaning from large gene/protein lists [22, 23], was used to identify KEGG pathways enriched in resveratrol-targeted genes. KEGG pathways with p<0.05 were selected for subsequent analysis. KEGG pathways enriched in both osteoporosis and resveratrol-targeted genes were visualized using a Venn diagram (Venny 2.1, http://bioinfogp.cnb.csic.es/tools/venny/index.html).

### Identification of hub genes

Enrichment information for the top five KEGG pathways was presented with GOplot, an R package that visually combines expression data with functional analysis [24]. Genes involved in all top five shared KEGG pathways were considered hub genes. Centrality in the network and chromosome position for all resveratrol-targeted genes were visualized using the circlize package for R [25].

### Retrieval of KEGG pathways related to resveratrol-targeted genes

The top five shared KEGG pathways with the smallest p values were selected, and KEGG pathways related to resveratrol-targeted genes were established, using Pathway Builder Tool 2.0 (http://www.proteinlounge.com). Hub genes and their mechanisms of action are shown in the schematic diagrams.

### Cell culture and transfection

BMSCs were kindly donated by the Huazhong University of Science and Technology, Wuhan, China. Cells were grown in a specific media designed for C57BL/6 mouse mesenchymal stem cells (#MUBMX-03011-440, Cyagen, Guangzhou, China) at 37°C in a 5% CO_2_ incubator. Cells were maintained for a maximum of 3 passages. Lipofectamine 3000 (#L3000001, ThermoFisher Scientific, USA) was used to transfect cells with siRNAs according to provided directions. MDM2 siRNA constructs (RIBOBIO, Guangzhou, China) were transfected at 50 nM. Plasmid NC and plasmid MDM2 were synthesized by GenePharma company (Shanghai, China). All *in vitro* experiments using cells were independently repeated three times.

### qRT-PCR analysis

Total tissue/cell RNA was extracted using Trizol reagent (#15596018, Invitrogen, USA). RNA was reverse transcribed using the Verso^TM^ cDNA Synthesis Kit (#AB-1054/A, ThermoFisher Scientific), and qRT-PCR reactions were performed using a Thermal Cycler C-1000 Touch system (#10021377, Bio-Rad CFX Manager, USA). GAPDH was used as a housekeeping gene. Target gene expression was quantified using the ΔΔCT method. The primers used in this study are listed in Table 2.

**Table 2:** Primers used in the experiments.

**Gene name**	**Primer sequence**
hsa - p53 - Forward	CAGCACATGACGGAGGTTGT
hsa - p53 - Reverse	TCATCCAAATACTCCACACGC
hsa - MDM2 - Forward	GAATCATCGGACTCAGGTACATC
hsa - MDM2 - Reverse	TCTGTCTCACTAATTGCTCTCCT
hsa - ALP - Forward	ACCACCACGAGAGTGAACCA
hsa - ALP - Reverse	CGTTGTCTGAGTACCAGTCCC
hsa - OCN - Forward	CAAAGGTGCAGCCTTTGTGTC
hsa - OCN - Reverse	TCACAGTCCGGATTGAGCTCA
hsa - Runx2 - Forward	TGGTTACTGTCATGGCGGGTA
hsa - Runx2 - Reverse	TCTCAGATCGTTGAACCTTGCTA
hsa - GAPDH - Forward	CCGTTGAATTTGCCGTGA
hsa - GAPDH - Reverse	TGATGACCCTTTTGGCTCCC

### Western blotting

Lysis buffer (#AS1004, Aspen, South Africa) containing 1% protease inhibitor (#AS1008, Aspen) was used to lyse cells and tissue samples. Proteins were separated via SDS-PAGE, transferred to NC membranes (#IPVH00010, Millipore, USA), blocked with 5% nonfat milk, and stained overnight at 4°C with antibodies specific for ALP (1:1000, Sigma, USA,#ab95462), Osteocalcin (1:500, Sigma, USA, #ab93876), RunX2 (1:500, Sigma, USA, #ab23981), *p53* (1:1,000, Sigma, USA, #SAB1302059), and GAPDH (1:10,000, Sigma, USA, #ab37168). Blots were then stained with appropriate horseradish peroxidase (HRP)-conjugated secondary antibodies (#AS1058, Aspen) and proteins were detected with a chemiluminescence detection system. Each experiment was independently repeated three times.

### Alizarin red staining

BMSCs were grown in 6-well plates in media containing 100 nM dexamethasone, 50 mM ascorbic acid, and 10 mM b-glycerophosphate to promote osteogenesis (#HUXMA-90021, Cyagen, USA). Briefly, cells were washed twice with PBS and 10% formalin was added to fix the cells for 15 minutes. Subsequently, 1 mL 0.5% alizarin red staining solution was used to stain the cells at room temperature for 15 minutes. After rinsing with distilled water for 5 minutes, red mineralized nodules were analyzed via a charge-coupled device microscope. Absorbance was measured at 570 nm. Experiments were repeated in triplicate.

### Statistical analysis

**Figure 8:**
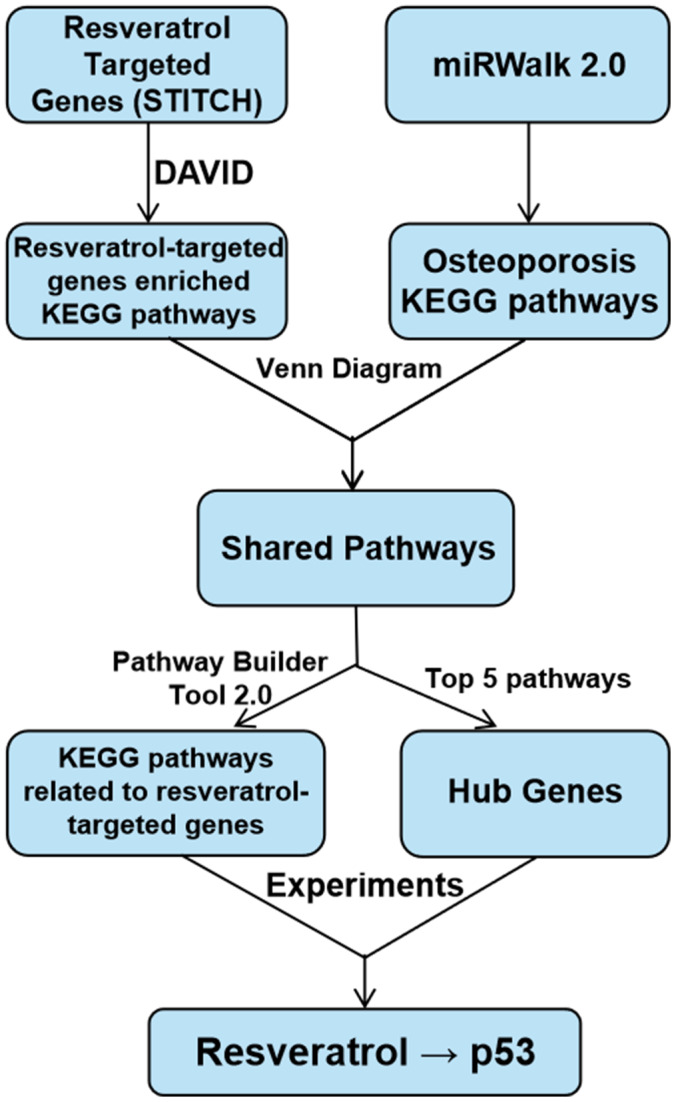
**Study schema.**

Data are shown as means **±** SD. GraphPad Prism 8.0 (GraphPad Software, CA, USA) was used to perform statistical analyses. One-way analysis of variance (ANOVA) with Tukey’s post hoc test was used to compare three or more groups. Unpaired Student’s t-test was used to compare data between two groups. P < 0.05 was considered statistically significant. The study schema is shown in Figure 8.
